# Anatomical Landmarks of the Facial Artery and Vein for Intraoral Anastomosis: A Cadaveric Study

**DOI:** 10.1002/micr.70004

**Published:** 2024-11-29

**Authors:** Kengo Nakatsuka, Tomoyuki Yano, Takuya Omotehara, Shinichi Kawata, Masahiro Itoh

**Affiliations:** ^1^ Department of Plastic and Reconstructive Surgery Cancer Institute Hospital of the Japanese Foundation for Cancer Research Tokyo Japan; ^2^ Department of Anatomy Tokyo Medical University Tokyo Japan; ^3^ Department of Anatomy Juntendo University Tokyo Japan

## Abstract

**Background:**

Intraoral anastomosis is a widely used technique for microvascular alveolar ridge augmentation and midface reconstruction. However, the predictable anatomical positioning of facial structures, such as the vessels, parotid duct, and facial nerve in the buccal region, has remained unclear. Therefore, we aimed to obtain the anatomical characteristics of these locations to establish surgical landmarks for the intraoral anastomosis of facial vessels.

**Methods:**

A total of 26 sides from 13 formaldehyde‐fixed cadavers approximately a month after fixation with a mean age at death of 86.6 ± 11.2 years (range: 55–104 years) were anatomically examined. Facial vessels, nerves, and the parotid duct were dissected intraorally. From the oral cavity side, the X‐axis was defined as the line from the labial commissure to the lowest point of the intertragic notch.

**Results:**

From the oral cavity side, all branches of the facial nerve were found under the facial artery and vein. The positioning order along the X‐axis was the facial artery, vein, and parotid duct exit. The facial artery was 21.3 ± 2.2 mm and the facial vein was 39.2 ± 2.7 mm from the labial commissure. Ninety‐two percent of facial veins were found within 15–20 mm of the facial artery on the X‐axis. The parotid duct exit was 46.8 ± 2.0 mm from the labial commissure. In the buccal region, the vessel calibers of the facial artery and vein were 1.8 ± 0.2 and 2.1 ± 0.2 mm, respectively.

**Conclusion:**

Knowledge of the anatomical relations among the facial artery, vein, parotid duct, and facial nerve from the oral cavity side can enhance the safety and efficacy of midface reconstruction surgeries involving intraoral anastomosis procedures.

## 1 Introduction

Since the introduction of intraoral anastomosis of free flaps in 2009 for microvascular alveolar ridge augmentation (Gaggl et al. 2009), intraoral anastomosis has been widely used for midface reconstruction, with favorable outcomes (Zheng et al. 2019; Wu et al. 2018). The advantages of intraoral anastomosis include favorable esthetic results and short pedicle flaps (Bueno de Vicente et al. 2021). Several papers have described methods for intraoral anastomosis using facial vessels running in the buccal region; however, these reports are limited to anatomical results (Gaggl et al. 2012; Brandtner et al. 2015; Stalder et al. 2017). This is because unlike anastomosis of familiar cervical vessels, or the submandibular identification of facial vessels, there is no consensus regarding the region of the buccal mucosa that should be incised to identify the vessels (Sosin et al. 2015). In addition, because of the lack of familiarity with the technique, the maximum size of a vessel that can be secured remains unknown. There are also concerns that the parotid duct or facial nerve may be damaged by performing the procedure from the buccal mucosa side. Conversely, if these points are clarified, there may be potential new uses for this technique.

The purpose of this study was to clarify the location of the facial artery, vein, parotid duct, and facial nerve, by performing anatomical dissections, thereby providing precise and reliable data that will be useful for future surgery, and to define clinical landmarks for use in surgical procedures.

## 2 Materials and Methods

A total of 26 sides of 13 formaldehyde‐fixed cadavers (five male and eight female cadavers) approximately a month after fixation with a mean age at death of 86.6 ± 11.2 years (range: 55–104 years) were examined for this study at Tokyo Medical University. This study was approved by the Institutional Review Board of Tokyo Medical University (study approval no.: T2020‐0050) for using cadavers.

**FIGURE 1:**
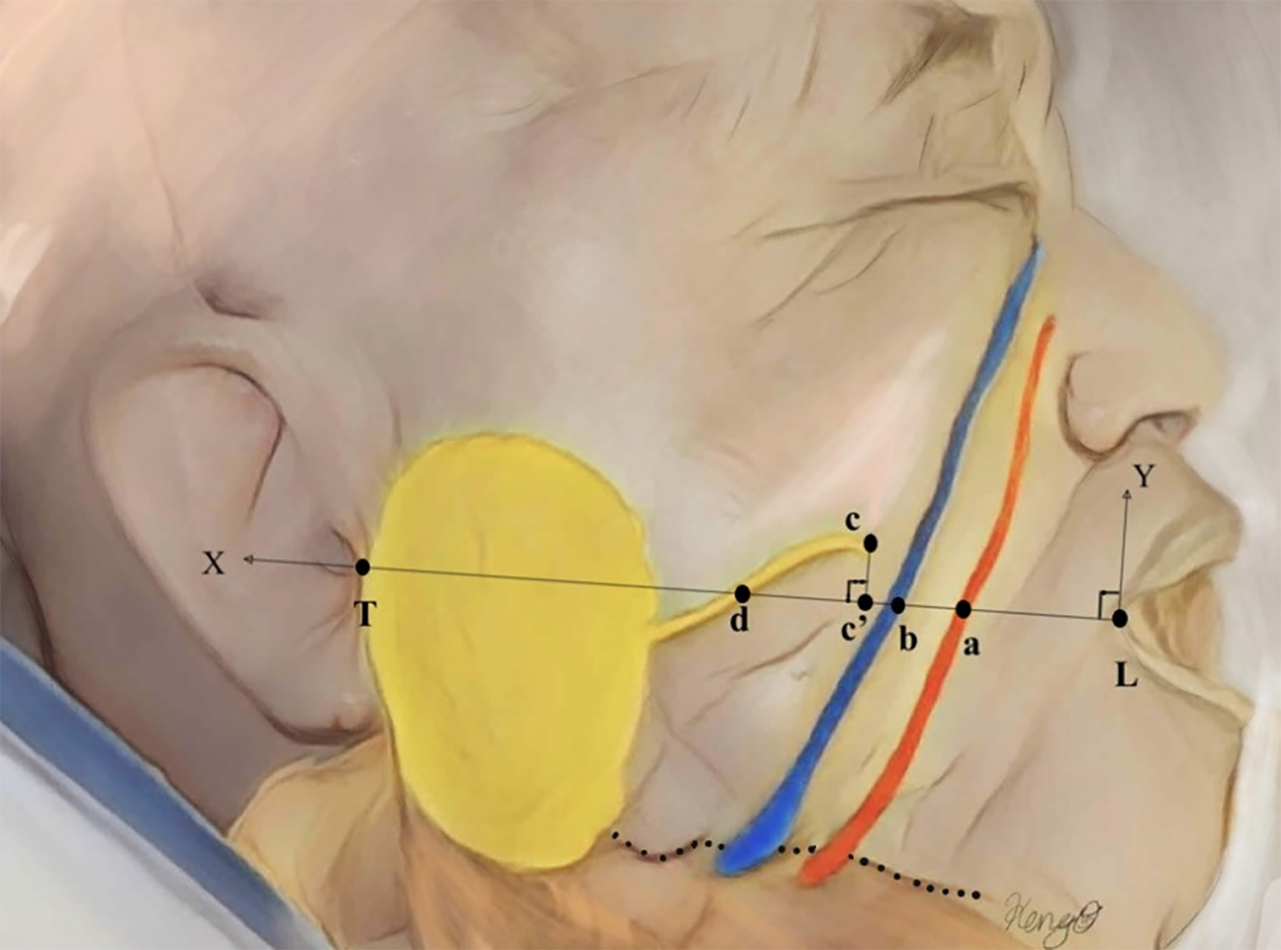
A Schema indicating reference Line 1 as an X‐axis and several indicative points were used in this study. The facial artery is drawn in red, the facial vein is drawn in blue, and the parotid gland and duct are drawn in yellow. The dotted line indicates the jawline. The X‐axis was defined as the line between the labial commissure and the lowest point of the intertragic notch, and the Y‐axis was defined as the line making a right angle with the X‐axis. The indicative points are as follows: (L) the labial commissure; (T) the lowest point of the intertragic notch; (a) where the facial artery crosses Line 1; (b) where the facial vein crosses Line 1; (c) the parotid papilla; and (d) where the parotid duct crosses Line 1. Because of the parotid duct having a curved course and point (c) consistently being above Line 1, point (c') was defined as a vertical projection of point (c) on Line 1. Various distances using those indicative points were measured from the oral cavity side (Table 1).

Facial vessels, facial nerves, and the parotid duct exit were carefully dissected in each hemiface from the oral cavity side. Regarding the measurements, the X‐axis was intraorally defined as Line 1 from the labial commissure (L) and the lowest point of the intertragic notch (T), and the Y‐axis was defined as the vertical line with the X‐axis from the L (Figure 1).

By using Line 1, various indicative points were marked on the line from the oral cavity side. First, the (L) and the (T) were marked with pins, and Line 1 was drawn by tying strings between these two pins. Second, several points were marked on Line 1 where the facial artery (point a), the facial vein (point b), and the parotid duct (point d) cross. Considering that a point of the parotid papilla, the exit of the parotid duct into the oral cavity (point c), is normally above Line 1, the vertical projection of point c on Line 1 (point c') was also marked (Figure 1).

**FIGURE 2:**
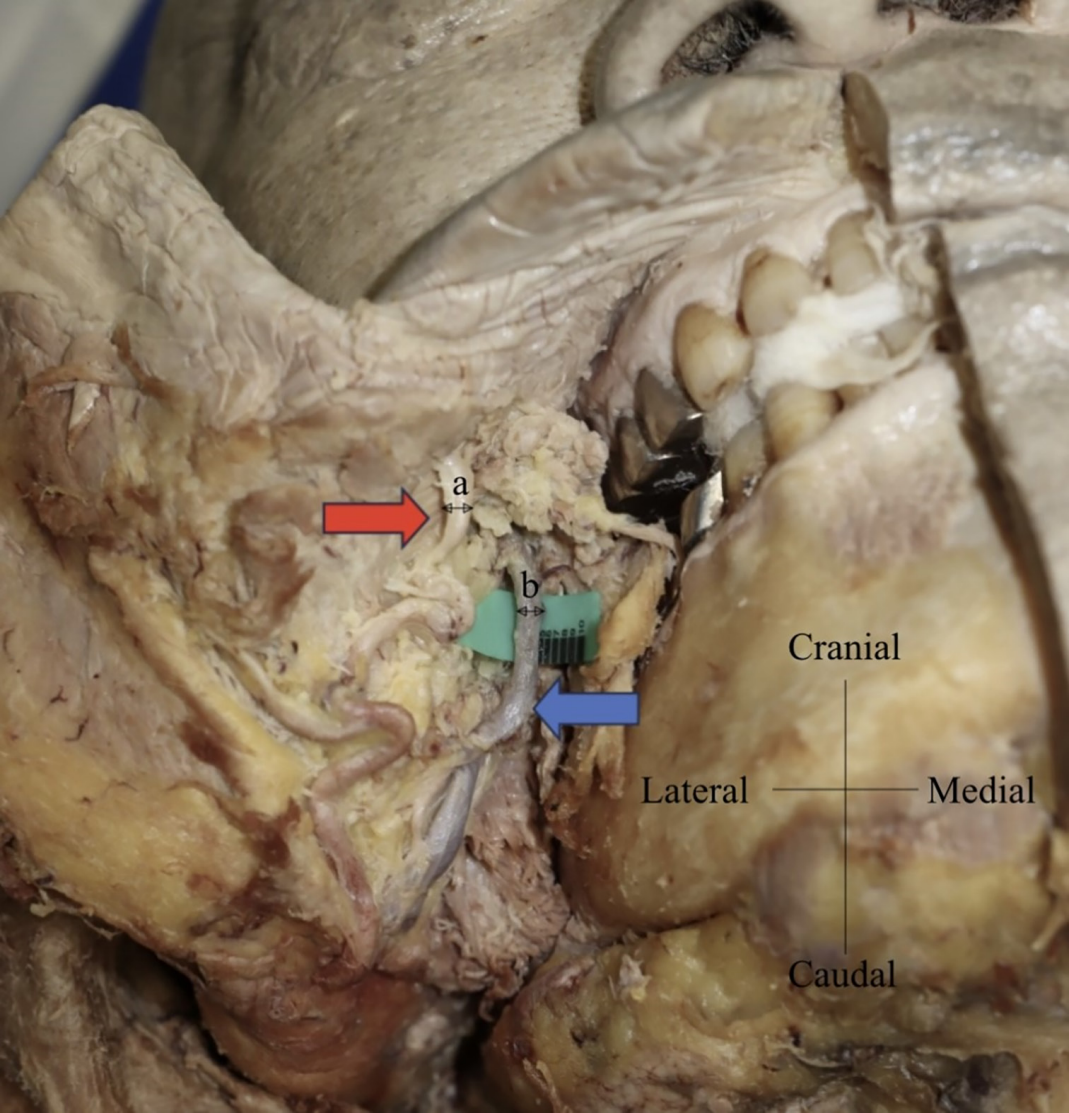
Autopsy of the facial artery and vein from the oral cavity side. The facial vessels were detected from the intraoral side. The red and blue arrows indicate the facial artery (a) and the facial vein (b), respectively. The vessel calibers of the facial artery and vein crossing Line 1 were measured (Table 2).

Data were obtained from measurements of the distance between the (L) and the (T) (Line 1), the distance between the (L) and the facial artery on Line 1 (L–a), the distance between the (L) and the facial vein on Line 1 (L–b), the distance between the (L) and the vertical projection point of the parotid papilla on Line 1 (L–c'), the distance between the (L) and the parotid duct on Line 1 (L–d), the distance between the facial artery and the facial vein on Line 1 (a–b), and the distance between the parotid papilla and its vertical projection point on Line 1 (c–c'). Additionally, the vessel calibers of the facial artery and vein on Line 1 were measured (Figure 2). All parameters were measured using a digimatic caliper (Mitsutoyo Corp., Kanagawa, Japan) with 0.1 mm accuracy, and the data were expressed by mean ± SD.

## 3 Results

### 3.1 Localization of the Facial Artery, the Facial Vein, and the Parotid Papilla on Line 1 From the Oral Cavity Side

All facial arteries and veins were found under the buccal mucosa and buccal muscles, and buccal branches of the facial nerve ran under the facial artery and vein in each cadaver. On Line 1 from the labial commissure, the facial artery (point a), the facial vein (point b), and the vertical projection of the parotid papilla (point c') were present in this order in all examined cadavers. Furthermore, the parotid papilla (point c) was consistently above Line 1.

### 3.2 Length of Line 1, and the Distances From the (L) to Points a, b, c', and d by Measurement From the Oral Cavity Side

The length of Line 1, extending from the (L) to the (T), was 107.7 ± 3.9 mm. The distance between the (L) and the facial artery (L–a) was 21.3 ± 2.2 mm. The distance between the (L) and the facial vein (L–b) was 39.2 ± 2.7 mm. The distance between the (L) and the vertical projection of the parotid papilla point on Line 1 (L–c') was 46.8 ± 2.0 mm. The distance between the (L) and the parotid duct on Line 1 (L–d) was 54.5 ± 1.4 mm. Furthermore, the distance between the facial artery and the facial vein on Line 1 (a–b) was 18.0 ± 2.5 mm. Ninety‐two percent of the facial vein was found between 15 and 20 mm from the facial artery on Line 1. The distance between the parotid papilla and its vertical projection on Line 1 (c–c') was 4.8 ± 1.8 mm (Table 1). Additionally, the vessel calibers of the facial artery and vein on Line 1 were 1.8 ± 0.2 and 2.1 ± 0.2 mm, respectively (Table 2).

**TABLE 1:** Measurements of the position of the facial artery, facial vein, and parotid duct regarding from Line 1.

	Line 1	L–a	L–b	L–c'	A–d	a–b	c–c'
Range (mm)	102.0–113.0	18.0–25.0	35.0–45.0	44.0–52.0	52.0–57.0	11.0–24.0	1.0–7.0
Mean ± SD (mm)	107.7 ± 3.9	21.3 ± 2.2	39.2 ± 2.7	46.8 ± 2.0	54.5 ± 1.4	18 ± 2.5	4.8 ± 1.8

*Note:* Line 1: distance between the labial commissure and the lowest point of the intertragic notch. L–a: distance between the labial commissure and the facial artery. L–b: distance between the labial commissure and the facial vein. L–c': distance between the labial commissure (L) and the right‐angle projection of point c on Line 1. L–d: distance between L and the point where the parotid duct crosses Line 1. a–b: distance between the facial artery (a) and the facial vein on Line 1. c–c': distance between the parotid papilla (c) and the right‐angle projection of point c on Line 1.

Abbreviation: SD, standard deviation.

**TABLE 2:** Vessel calibers of the facial artery and the facial vein on Line 1.

	Vessel caliber	
	Facial artery	Facial vein
Range (mm)	1.5–2.2	1.8–2.4
Mean ± SD (mm)	1.8 ± 0.2	2.1 ± 0.2

Abbreviation: SD, standard deviation.

## 4 Discussion

In this anatomical dissection study on 26 hemifaces, we tried to make surgical landmarks of the facial artery, the facial vein, and the parotid duct from the oral cavity side using the transverse incision as a new incision line to approach the recipient vessels for intraoral anastomoses. The main finding of our study was to clarify the location of the facial artery, vein, parotid duct, and facial nerve, by performing anatomical dissections. Furthermore, this study detailed the size of facial vessels as for the recipient vessels. From this precise and reliable data, we made a new incision line to expose the facial artery and vein as recipient vessels from the oral side without damaging the parotid duct and facial nerve. This will be potentially useful for microsurgeons to safely perform intraoral microsurgery.

In regard to the facial artery, a previous study analyzed the location of facial arteries from the body surface and reported that the facial artery lies 19.4 mm from the oral commissure on a line between the oral commissure and the lowest point of the intertragic notch (Calva et al. 2015). Lee et al. also reported that the facial artery is located 16 mm from the oral commissure, on a line between the oral commissure and the lowest point of the earlobe (Lee et al. 2018). These measured values from the facial surface side seem a little shorter than those from the oral cavity side in the present study. Although previous papers have not mentioned the diameter of the facial artery in the same region, the present study confirmed that the diameter was 1.5–2.3 mm, which is thick enough to use as a recipient artery.

Clinically, when the facial artery is used as the recipient vessel, it should be examined preoperatively by palpation, Doppler ultrasound, and echocardiography to determine how it runs, since there are some exceptional cases in which the facial artery does not cross the incision line. In the present study, we were able to identify facial arteries on the incision line in all examined cadavers. However, Lohn et al. reported that 5% of the arteries did not terminate over the line between the labial commissure and the lowest point of the intertragic notch in 201 specimens (Lohn et al. 2011), and Koh et al. also reported that 4.5% of the arteries did not cross over the same line in 47 specimens (Koh et al. 2003).

Regarding the facial vein, Gaggl et al. found that the vein is located approximately 10 mm distal from the facial artery measured in the paramandibular region (Gaggl et al. 2009). In our study, the relative distance of the facial artery and vein was variable, but 92% of cases were within 15–20 mm. The course and the patterns of the facial vein are described as being quite predictable, with only rare variations ranging between 0% and 1.5% (Lohn et al. 2011; Cotofana et al. 2017). In all cadavers examined in the present study, we were able to identify all facial veins between the labial commissure and the lowest point of the intertragic notch. However, Lohn et al. mentioned the case where the facial vein was absent and was replaced by a transverse facial vein originating in the inner canthus and merging with the superficial temporal vein in the parotid gland (Lohn et al. 2011). As with facial arteries, preoperative testing is required for facial veins if they are to be used as recipient vessels. To our knowledge, no reports of the diameter of the facial vessels in the buccal region are available. The present study showed that the diameter of the facial artery was 1.8–2.4 mm, which appears to be adequate as a recipient vessel.

Additionally, all facial nerves examined were identified under both facial arteries and veins from the oral cavity side. Previous studies have also reported that the facial nerves lie deeper than the facial artery and vein (Cotofana et al. 2017; Ayad and Xie 2015). Therefore, there has been no report of postoperative neurological complications after intraoral anastomosis in previous studies (Zheng et al. 2019; Bueno de Vicente et al. 2021; Brandtner et al. 2015).

In a previous anatomical dissection study using 46 hemifaces, Uzmansel et al. used the same indicative line that extends between the labial commissure and the lowest point of the intertragic notch and reported that the position of the parotid duct opening was 30.2 mm from the labial commissure and 11.7 mm above Line 1 (Uzmansel, Elvan, and Aktekin 2022). They also documented that the distance of (L–d) was 53.9 mm (Uzmansel, Elvan, and Aktekin 2022). In another anatomical dissection study using 35 hemifaces, Toure et al. measured the distance between the labial commissure and the point across Line 1 of the parotid duct and gave it as located at the distal 71 mm (Toure, Foy, and Vacher 2015). Based on the present study showing that the distance of (L–d) was 54.5 mm, and previous reports in the literature, it is likely that there may be a racial differences; however, in general, the parotid duct does not intersect until 50 mm from the labial commissure, with Line 1 as the reference line (Uzmansel, Elvan, and Aktekin 2022; Toure, Foy, and Vacher 2015).

Introduced by Gaggl et al. 2009, the complete intraoral reconstruction technique avoids the formation of extraoral scars (Gaggl et al. 2009). Over time, the intraoral reconstruction technique has become an option requiring no extraoral incision for patients who have undergone intraoral resection and those who have an intraoral defect (Qiu et al. 2023; Garg et al. 2023; Landes et al. 2014). The method used in this technique to dissect the recipient vessels is basically the same as in previous papers (Gaggl et al. 2012; Brandtner et al. 2015; Stalder et al. 2017). After confirming the location of the facial artery by Doppler ultrasound and arterial pulsation preoperatively, an oblique incision is made anterior to the parotid duct exit to locate the artery and then the vein. However, previous studies have pointed out that although identifying facial arteries is easy, there are some difficulties in identifying facial veins. (Brandtner et al. 2015; Sosin et al. 2015) One reason for this is that facial veins are posterior to the facial arteries (Brandtner et al. 2015). According to the present study and other studies in the literature, the parotid duct does not cross until 50 mm from the labial commissure, with Line 1 serving as the reference line (Uzmansel, Elvan, and Aktekin 2022). By keeping the incision line on Line 1, 50 mm from the labial commissure, the posterior view can be secured safely and the approach to the facial vein is facilitated.

**FIGURE 3:**
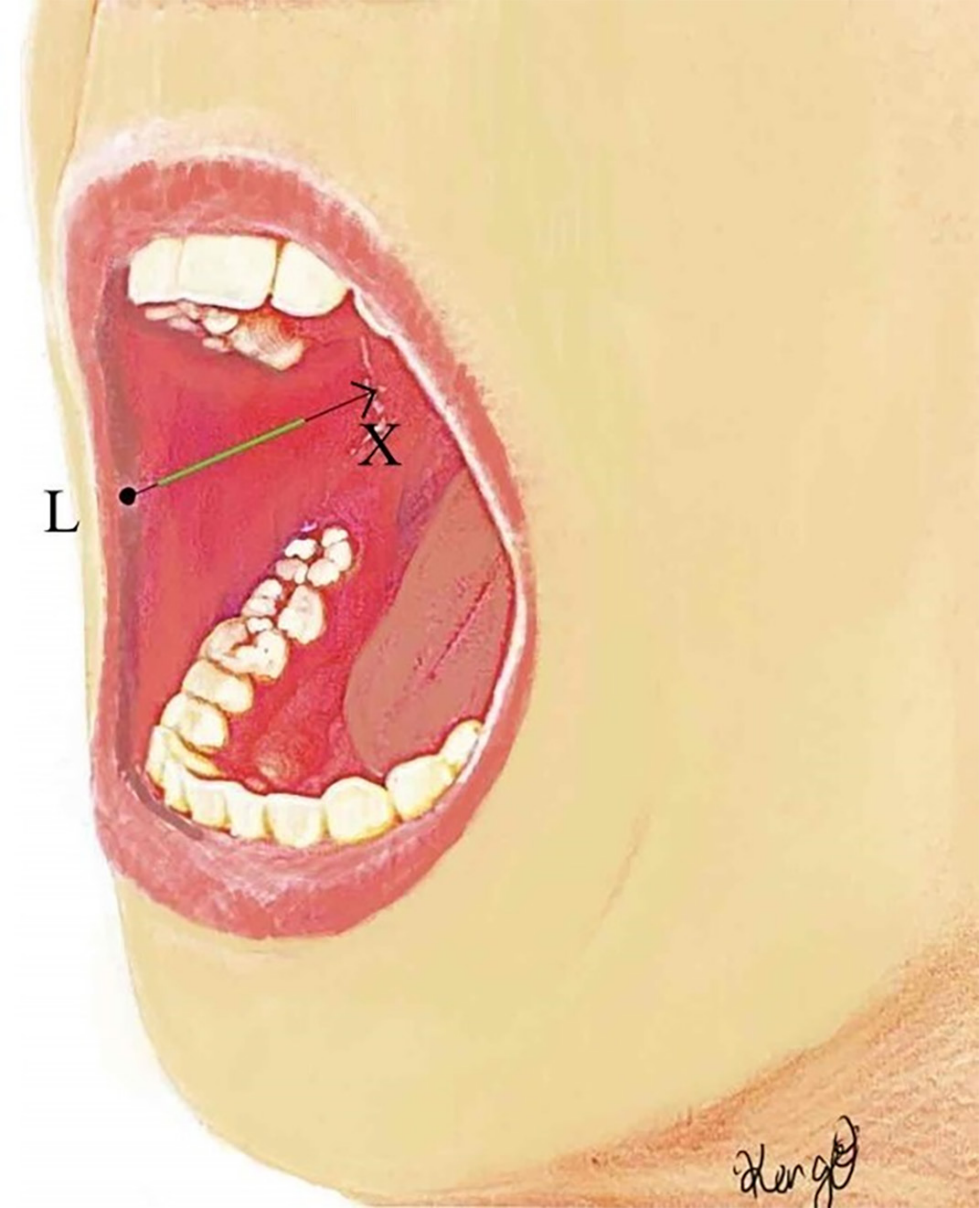
Schema showing the new incision line for approaching facial vessels. The X‐axis was defined as the line between the labial commissure and the lowest point of the intertragic notch. The point (L) indicates the labial commissure. The green line mentions the new incision line 1.5–5 cm from the oral commissure on the X‐axis.

**FIGURE 4:**
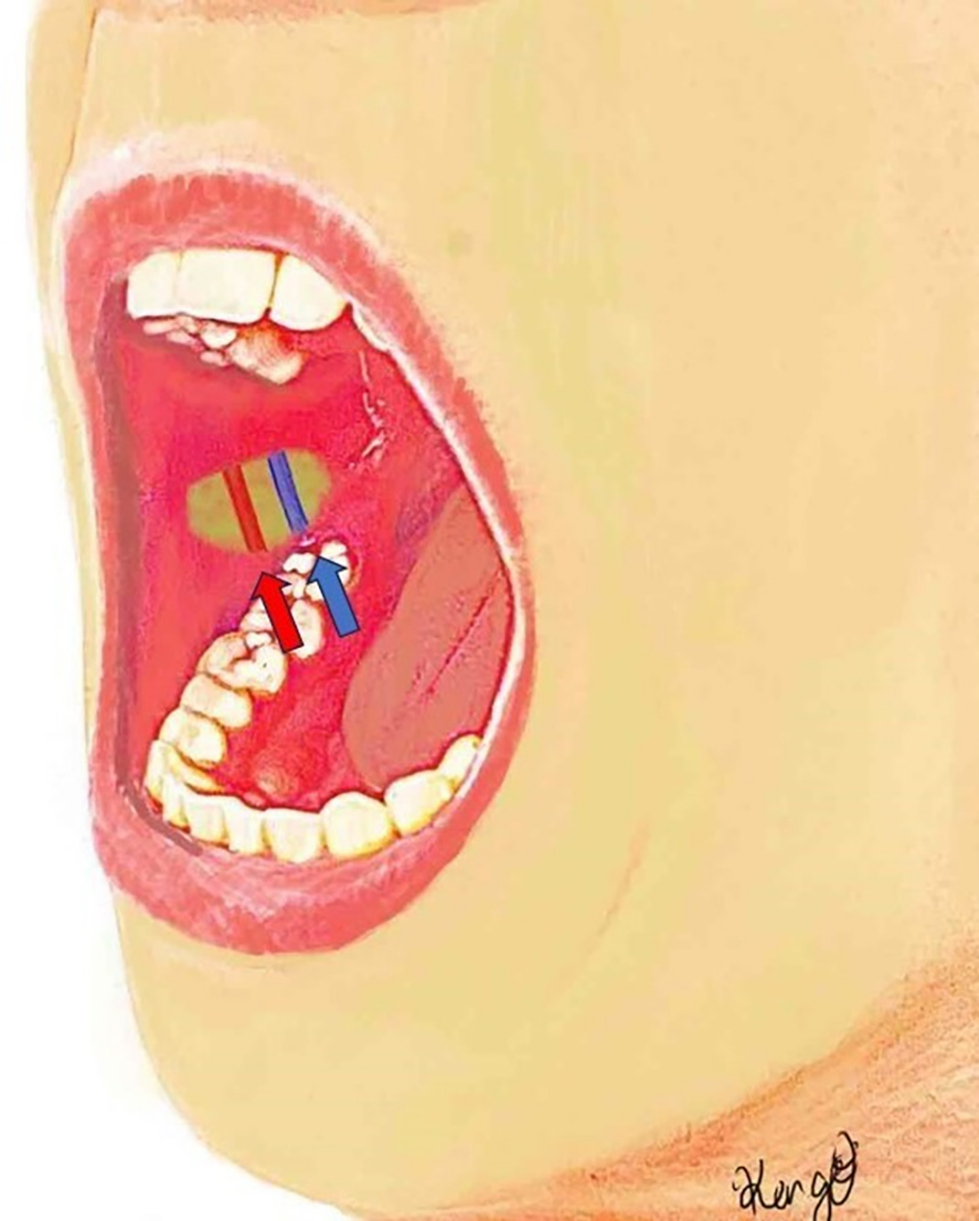
Schema showing facial vessel dissection from the new incision line. The facial vessels were dissected from the intraoral side. The red and blue arrows indicate the facial artery and the facial vein.

Based on our data, we established a new incision line along the line connecting the labial commissure and the lowest point of the intertragic notch. By placing the incision 1.5–5 cm from the oral commissure along this line, we were able to safely expose the facial artery and vein as recipient vessels from the oral side without damaging the parotid duct (Figure 3). Additionally, it is important to note that if the dissection extends superiorly beyond the reference line, there is a risk of damaging the parotid duct. Therefore, when exposing the recipient vessels, it is crucial to ensure sufficient for anastomosis by maintaining the dissection on the caudal side (Figure 4).

There are several limitations to our study. First, it is true that errors due to some traction or unevenness of the buccal area may occur when measuring. With this in mind, we took care to ensure that the pull during measurement was the same each time. Second, anatomical data were obtained from cadavers that had been fixed in formaldehyde, thus, the facial tissues were stiff, contracted, less mobile, and shrunken to some degree compared with living patients. Furthermore, variations in the amount of fatty tissues and cheek thickness among the patients may have caused variations in the location of facial vessels. Clinically, when the facial vessel is used as the recipient vessel, it should be examined preoperatively by echocardiography to determine how it runs.

## 5 Conclusions

The facial artery and vein were identifiable from the oral cavity side in all cadaver specimens on the line between the labial commissure and the intertragic notch. Although there is variability of the running courses of the facial artery, vein, and nerve among patients, the facial artery runs approximately 20 mm posterior from the labial commissure, and in 92% of the specimens, the facial veins were found on the line at 15–20 mm posterior from the facial artery. It also appeared that a risk of damage to the parotid duct can be avoided if the incision is made at less than approximately 50 mm from the labial commissure. The facial nerves lie deeper than the facial artery and vein from the oral cavity side. Therefore, it is expected that the results of the present study can be used to predict the location of these vessels and nerves before performing the intraoral reconstruction technique. However, it must be kept in mind that these anatomical data were obtained from cadavers that had been fixed in formaldehyde, thus, the facial tissues were stiff, contracted, less mobile, and shrunken to some degree compared with living patients.

## Ethics Statement

This report was published with the consent and permission of the patients involved.

## Conflicts of Interest

Tomoyuki Yano is an Editorial Board member of Microsurgery and a co‐author of this article. To minimize bias, he was excluded from all editorial decision‐making related to the acceptance of this article for publication.

## Data Availability

The data that support the findings of this study are available from the corresponding author upon reasonable request.
